# The Modelling of Convective Drying Variables’ Effects on the Functional Properties of Sliced Sweet Potatoes

**DOI:** 10.3390/foods11050741

**Published:** 2022-03-02

**Authors:** Elif Savas

**Affiliations:** Department of Food Engineering, Faculty of Engineering, Balıkesir University, Balıkesir 10143, Turkey; esavas@balikesir.edu.tr; Tel.: +90-266-612-1194

**Keywords:** freeze drying, convective drying, response surface methodology, sweet potato, shrinkage

## Abstract

Drying is a commonly used technology that provides a long post-harvest storage time for produce such as sweet potatoes. Convective drying (CD) is a method that, when conditions are optimized, provides produce with a better appearance and improved textural properties. In this study, changes in water activity (a_w_), moisture content (MC), rehydration capacity (R_c_), shrinkage (S_b_) and color attributes were modelled for the optimization of drying factors (temperature, thickness and time) using response surface methodology (RSM). The total phenol content (TPC), total flavonoid content (TFC), total anthocyanin content (TAC) and antioxidant activity of CD samples (63.79 °C, 4.78 h, 3 mm) were investigated as functional aspects and compared with results for FD samples (−45 °C, one term). Optimum convective drying conditions caused an increase in general antioxidant properties, such as total phenol (TPC), total anthocyanin (TAC), DPPH and CUPRAC. The TPC was 190.94 mgGAE/100 g, the DPPH scavenging activity was 12.05%, the TAC was 11.37 mg/100 g, and the CUPRAC was 0.469 mmolTR/g in convectively dried samples under optimum conditions. Although improved appearance and textural properties are obtained by freeze drying, it is possible to produce sweet potatoes with good appearance and functional properties by optimizing the variables of the convective drying process.

## 1. Introduction

Sweet potato (Ipomea batatas) is among the most widely produced food sources in developing countries after grains. These tubers, of which 90 million tons are produced annually in Asian countries and more than 105 million metric tons globally, are used in the production of sweet bread and bakery products [1]. There are different types, ranging from white to yellow, orange and light purple according to the color of the skin and flesh. Depending on the variety, sweet potato has been reported as a rich food source with B, C, E and K vitamins; dietary fiber; phosphorus; potassium; and calcium content [2]. Purple sweet potatoes attract attention due to their high anthocyanin content, while the orange variety is of interest due to its anticarcinogenic beta carotene content. Sweet potato is ranked among the best root and tuber vegetables with its high antioxidant content and antidiabetic effect [3]. The perishable roots are mostly stored as flour, produced by the drum drying method, or as slices, processed by the convective drying method [4].

Drying is a food processing method that has been used safely for centuries for the long-term storage of fruits and vegetables [5]. Drying methods aim to remove most of the moisture from food with minimal cost and maximal efficiency [6]. Convective drying is a conventional method based on moisture evaporation with heat treatment to prevent the growth of microorganisms [7]. The water activity reduction caused by moisture loss increases the losses of sugar, protein, color substance and functional components, which are sensitive to heat. The functional components’ CD heat sensitivity can be protected at low temperatures without deformation—in fact, depending on the product matrix, a tendency for high antioxidant activity may occur [8].

Color attributes and shrinkage values in the drying process are among the most important appearance properties affected by process conditions [9]. Sensory attributes such as dark color formation, low rehydration capacity and a high hardness value have been reported in hot air-dried vegetables and fruits. Furthermore, positive effects of heat treatments on the functional properties of foods containing beta carotene and flavonoids have been reported [10]. In dried products, the expectation is mostly the preservation of appearance, texture and functional properties. Factors such as a change in the products dried via the convective drying process and the demand for green energy have revealed the need to use different drying methods [11]. It is thought that the current method can be used as a more efficient and environmentally friendly method when the process factors are optimized. Freeze drying is considered a safe method for the drying of fruits and vegetables without heat damage to their sensorial properties. The ice-sublimation leads to reductions in water activity and moisture content without damaging the texture and color attributes in the food matrix. The best texture and color scores were reported for freeze-dried samples compared to other drying methods, such as hot air, microwave and vacuum drying [12].

The antioxidant properties of dried sliced sweet potato have been presented in previous studies using different methods (convective, microwave and vacuum-freeze drying) at selected fixed temperatures or slice thickness [13,14]. The drying factors in these studies were not optimized to investigate the best drying conditions in terms of antioxidant or physico-chemical properties [15]. The drying process carried out under optimized conditions will inspire further studies in terms of preserving the antioxidant properties of the product, obtaining better appearance and texture properties. This process aims to increase time, energy and product efficiency by optimizing the production conditions by using modeled results. Response surface methodology is known as a safe method for modelling the linear, quadratic and interactive effects of variable responses with BBD for the optimization of conditions in food processes.

In this study, the effects of convective and freeze-drying processes on the physicochemical and functional properties of sliced sweet potatoes were investigated. Linear, quadratic and interactive effects of temperature, time and slice thickness on the color, texture and antioxidant activity of sweet potatoes were determined in the CD process. The optimum conditions for the CD were determined using response surface methodology (RSM) with the Box-Behnken design. Experimental data obtained under optimum conditions were compared with freeze drying results.

## 2. Materials and Methods

### 2.1. Sample Preparation and Drying Processes

All of the chemicals used for analysis were of analytical grade (Merck, Darmstadt, Germany). The tubers of sweet potato were obtained from a local market (Tokat, Turkey). The unpeeled potato tubers after washing were sliced into thicknesses of 3.00, 4.5 and 6.00 mm to ensure as uniform a heat transfer as possible. Slices of as similar a size as possible were dried using a hot-air dryer (SDE-S6780, Sedona, Korea) in stainless-steel trays (250 mm × 330 mm) at 4.1 m/s of air velocity, with 10% of relative humidity. The air velocity and relative humidity were measured using a Thermo-Anemometer (Model 451104, Extech Instruments, Taiwan) coupled with a T-type thermocouple (THD-R, Autonics, Malaysia). The frozen sweet potatoes sliced at 4.00 mm thickness were freeze dried at −45 °C for freezing and +48 °C for final drying, 0.44–2.8 MPa, 6CFM of flow rate using a freeze drier equipped with a vacuum pump (Harvest Right, LLC. Shanghai, China). The dried sweet potatoes were stored in moisture and oxygen-proof packages at −20 ± 2 °C temperature.

### 2.2. Experimental Design and Data Analysis

The experimental procedure was designed for the convective drying process using RSM with the Box–Behnken design to investigate the individual and quadratic effects of the variables defined as temperature (X_1_), thickness (mm) (X_2_) and time (hour) (X_3_) on the physico-chemical and functional properties of sweet potatoes. The following polynomial regression equation including the main, quadratic and interaction effects on the response surface was used to observe the variables (Equation (1)):

Y = β_0_ + β_1_X_1_ + β_2_X_2_ + β_3_X_3_ + β_4_X_1_X_2_ + β_5_X_1_X_3_ + β_6_X_2_X_3_ + β_7_X_12_+ β_8_X_22_ + β_9_X_32_ (1)

where Y is the response estimated from the model; β_0_, β_1_, β_2_, β_3_, β_4_, β_5_, β_6_, β_7_, β_8_, and β_9_ are the regression coefficients of constant, linear, quadratic and interaction effects; and X_1_, X_2_, X_3_ represent the independent variables for temperature, thickness and time, respectively. An analysis of variance (ANOVA) was carried out to assess the predicted model on the response variable for each factor at a 95% confidence level using Minitab^®^ statistical software (State College, PA, USA). The fitness of the regression model was determined using the regression coefficient (R^2^), *p*-values of the lack of fit (LOF) and a regression model. Optimum conditions were determined using 3D plots representing the response surfaces.

Validation of the optimized conditions was generated for minimum water activity (a_w_), moisture content (MC) and shrinkage (S_b_) as well as for the maximum total phenol content (TPC), total anthocyanin content (TAC), total flavonoid content (TFC), antioxidant activity (DPPH) and antioxidant capacity (CUPRAC) values. Responses were obtained under the optimized drying conditions. The model was validated by comparison with the estimated and experimental value based on CV%.

### 2.3. Physicochemical Attributes

The oven drying method was used to determine moisture content (MC) until a constant mass was obtained. The results were calculated using following equation:

(2) $$\mathrm{MC}\;=\;(W_{1}-W_{2})/(W_{2}-W_{3})\;\times \;100$$

where the initial weight of the weigh bucket and the sample is expressed as W_1_, the final weight of the weigh bucket and the sample is expressed as W_2_, and the weight of the weigh bucket is expressed as W_3_.

The water activity (a_w_) of the samples was measured (0.002 accuracy) using a water activity meter (FastLab, GBX Sci. Ltd., Dublin, Ireland) calibrated with K_2_SO_4_ standard solution (a_w_ = 0.986).

The shrinkage (S_b_) of dried and undried sweet potato samples was determined by measuring change in volume. Firstly, the volumes of randomly selected sliced potatoes were measured by toluene displacement. The following equations were used to calculate shrinkage and apparent density (ρ_a_) of SP:

(3) $$S_{b}=\frac{V_{0}-V}{V}\;\cdot \;100$$

(4) $$\rho_{a}=\frac{m}{V}$$

where S_b_ is shrinkage (%); V_0_ and V are the initial and final volumes of SP (cm^3^), respectively; ρ_a_ is the apparent density (g/cm^3^); m is the potato mass (g); ρ_b_ is the bulk density; and X_w_ is the water mass in SP (Equation (5)):

(5) $$\rho_{b}=\frac{1}{\frac{X_{w}}{1000}+\frac{1-X_{w}}{1590}}$$

The dry samples were weighed and placed into a glass baker containing 150 mL of distilled water at room temperature for 6 h to measure rehydration capacity. The samples were placed on clean and dry filter paper to remove the water. The following equation was used for estimation of the rehydration capacity [16]:

(6) $$R_{c}=\frac{m}{m_{0}}$$

In order to determine the browning value (B_v_) of the SP, after rehydration, water was clarified by centrifugation at 3200× *g* for 10 min, and an equal volume of ethanol (95%) was added into the supernatant. The mixture was centrifuged again at 3200× *g* for 10 min. The B_v_ was determined, measuring absorbance at 420 nm using a UV-visible spectrophotometer (T80+; PG Instruments, Wibtoft, UK) [17].

### 2.4. Texture Analysis

The texture properties were determined by measuring the maximum tolerable force, which is the hardness criterion of the dried sweet potatoes, using a texture analyzer (TA-XT, Texture Technologies Corp, Godalming, Surrey, UK). Hardness was the peak force of the first compression cycle in N, cohesiveness was the positive force area during the second compression compared to that during the first compression, springiness was the time duration of force input during the second compression compared to that during the first compression, resilience was the negative force input compared to positive force input during the first compression, adhesiveness was the negative area under the baseline between the compression cycles, and chewiness was the hardness multiplied by cohesiveness multiplied by springiness in N.

### 2.5. Color Attributes

The color properties in a three-dimensional space of the fresh and dried SP were analyzed using a spectrocolorimeter (LC100, Lovibond, Maharashtra, India). The L* value as the lightness, the a* value as the redness, and the b* value as the yellowness were measured at room temperature. The instrument was calibrated using a white tile in line with the manufacturer’s recommendations. Chroma (saturation) and the Hue (H) angle were calculated as follows:

(7) $$\mathrm{Chroma}=\sqrt{\left(a^{2}+b^{2}\right)}$$

(8) $$\mathrm{Hue}\;\mathrm{angle}=\tan^{-1}+\left(\frac{b}{a}\right)$$

### 2.6. Microstructure Analysis

A scanning electron microscope (SEM) (Quanta, 250 FEG FEI Co., Eindhoven, The Netherlands) was used to determine drying effects on the microstructure of dried samples. A very thin layer of gold was coated on the sample surfaces (thickness 0.1 cm) to obtain clear SEM images [16].

### 2.7. Thermal Properties

The thermal properties of dried potatoes were determined using a differential scanning calorimeter (Hitachi High-Tech Sciences DSC7020, Tokyo, Japan). After a 3–5 mg pellet was sealed hermetically in an aluminum pan, the heating from 40 to 75 °C at a rate of 2 °C/min was processed. The reference was an empty pan. Thermal properties were recorded as the temperature of onset, peak, conclusion and enthalpy change [17].

### 2.8. Antioxidant Activity

In order to perform the extraction of antioxidants from SP, firstly, 0.5 g of the samples were placed into a 50 mL centrifuge tube with 15 mL of 80% methanol and were vortexed for 30 s. After shaking the mixture at room temperature for 2 h, it was centrifuged at 3000× *g* for 10 min. The supernatant formed after centrifugation was used in the antioxidant assay [18].

The total antioxidant activity (TAA) of SP was assessed using the 2,2-diphenyl-1-picrylhydrazyl (DPPH) method [19], with some modifications, using a UV-Vis spectrophometer (T80+; PG Instruments, Wibtoft, UK) at 517 nm. The results, expressed as µmol of Trolox equivalent per gram of the sample (µmol TE/g), were calculated from the calibration curve (R^2^ = 0.9972) plotted using different amounts of Trolox (10–1000 µmol/L).

The cupric-reducing antioxidant capacity (CUPRAC) was performed as described previously by Abuduaibifu and Tamer [19] with slight modification and was expressed as µmol Trolox equivalent (TE) per g of sample. The calibration curve was plotted (R^2^ = 0.9899) using different amounts of Trolox (10–500 µmol/L).

The total anthocyanin content (TAC) was estimated using a modified method as defined by Jiang et al. [20]. The measurement of absorbance was performed at 527 and 700 nm using spectrophotometer (UV-Vis, T80+; PG Instruments, UK), while distilled water was used as a blank. The results were expressed as cyanidin-3-glucoside equivalent:

(9) $$\mathrm{TAC}\;\left(\frac{\mathrm{mg}}{g}\right)=\frac{A}{\varepsilon L}\;\times \;\mathrm{MV}\;\times \;\mathrm{DF}\;\times \;\frac{V}{m}$$

where A = (A_527_ − A_700_)_pH 1.0_ − (A_527_ − A_700_)_pH 4.5_; MW is the molecular weight of cyanidin-3-glucoside (449.2 g/mol); V is the volume of the extract (mL); L is the cell path length (1 cm); DF is the dilution factor; ε is molar absorption coefficient of cyanindin-3-glucoside (26,900); and m is the weight of the sample (g). The modified method described by Yea et al. (2019) was used to determine the total flavonoid content. The absorbance was measured at 510 nm using a UV-vis spectrophotometer (T80+; PG Instruments, UK). The TFC value was expressed as milligrams of catechin equivalent per gram (mg CE/g) of the sample using a calibration curve plotted with different catechin amounts (0–300 mg/L).

The Folin–Ciocalteu method was used to determine the total phenolic content (TPC) with some modifications (Tayyab Rashid et al., 2020). The absorbance was determined spectrophotometrically (UV-Vis, T80+; PG Instruments, UK) at 760 nm. The TPC amount was expressed as milligrams of gallic acid equivalent per gram (mg GAE/g) of the sample.

## 3. Results

### 3.1. Response Surface Analysis

The effects of the independent variables on the MC, a_w_, L*, a* and b* values, chroma and hue, as well as the S_b_, R_c_, ρ_a_, ρ_b_, B_v_, TAC, TFC, TPC, DPPH and CUPRAC of the hot air-dried SP, are presented in Table 1. Interaction between dependent and independent variables was modelled using the quadratic polynomial equation (Equation (1)). The data showed that the fitted model can be used at 95% confidence level to predict the response surfaces of the three independent variables (Table 2 and Table 3).

**Figure 1:**
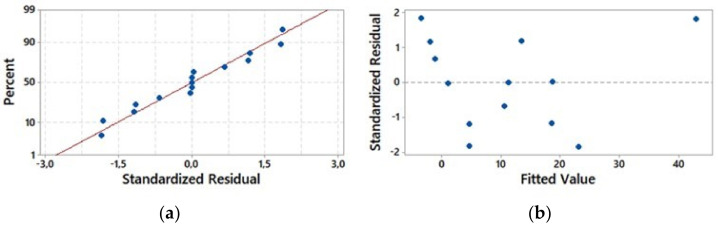
The normal probability (**a**) and fit plots (**b**) from the moisture content of hot air-dried sweet potatoes.

The adequacy of approximation in fitted models was examined using a real system. The residuals from the least squares fit were used to determine the adequacy of the models, and, also, the normality assumption were evaluated using normal probability plots by constructing the residuals against normal present probability (Figure 1a). The normality assumption for all responses was fulfilled, while the residuals formed a linear line in the normal probability plots (Figure 1b).

### 3.2. Physicochemical Properties

The determination of optimum drying conditions is a priority for the appropriate process design for the drying of fruits. Water activity (a_w_) and moisture content (MC), which are among the most important criteria for the shelf life of dried foods, are used to determine the drying efficiency (Table 4).

**Figure 2:**
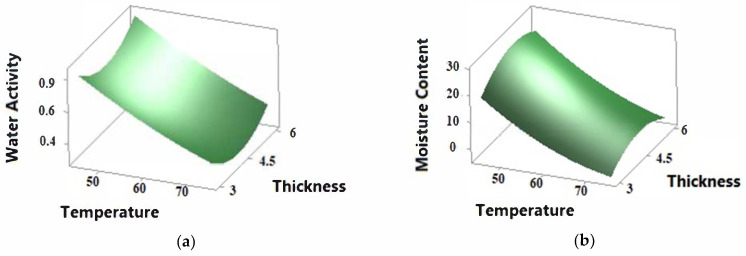
Surface plots of the (**a**) water activity and (**b**) moisture content of the hot air-dried sweet potatoes as affected by the drying conditions of temperature and thickness.

The 3D versus plots demonstrate the optimum values of MC and a_w_ for the convective drying of sweet potatoes (Figure 2a,b).

The MC of the SP ranged from 0.30 to 46.90% (Table 1). According to Table 3, the effect of temperature and time was significantly negative (*p* < 0.05) on MC, whereas that of thickness was slightly negative (*p* > 0.05). The three independent variables negatively affected a_w_, ranging from 0.431 to 0.934. Logarithmic equations gave the best fit of a_w_ with temperatures at different thicknesses. MC and a_w_ were exposed to the second order and quadratic negative effects of all factors, especially temperature.

The shrinkage characteristics consist of slice thickness as well as apparent and bulk densities in this study. The maximum volume reduction was 89.47% at 60 °C, 6 mm of thickness and after 6 h of drying time. The evident shrinkage was not determined in the freeze-dried potato samples. Time and thickness had a greater effect on q_a_ and q_b_ values than temperature.

### 3.3. Color Attributes

**Figure 3:**
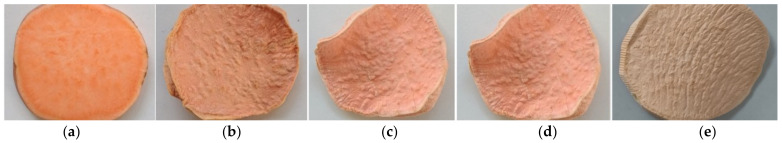
Sliced sweet potatoes: (**a**) fresh, (**b**) convective dried for 2 h, (**c**) convective dried for 4 h, (**d**) convective dried for 6 h, (**e**) freeze dried.

The effect of drying factors on the color characteristics is given in Table 1 and Table 3, and the model equations of the variables are given in Table 2. The highest L* values were measured for the convectively dried (60 °C, 6 h and 6 mm thickness) potato sample at 73.3, whereas for the fresh sample this value was 55.50. An increase in the temperature of the CD had a significant positive effect on the L*, b* and hue values and a significant (*p* < 0.05) negative effect on the a* value (Table 3). The L* values of the samples increased depending on the drying time. The discoloration of the samples losing moisture is clearly visible. Slice thickness and temperature were determined to be important factors affecting color characteristics (*p* < 0.05). The chroma values decreased from 43.5 in fresh products to 30.2 with freeze drying and to 33.66 with convection drying in optimum conditions. After drying, the SP appeared lighter, as observed from the higher L* and lower a* values as compared to the fresh SP (Figure 3). There was no significant effect among the drying factors and browning values of the CD samples. A higher L* value was also obtained in the freeze-dried samples compared to in the hot air-dried and fresh PS, whereas lower a* and b* values were determined (Figure 3e).

### 3.4. Antioxidant Properties

**Figure 4:**
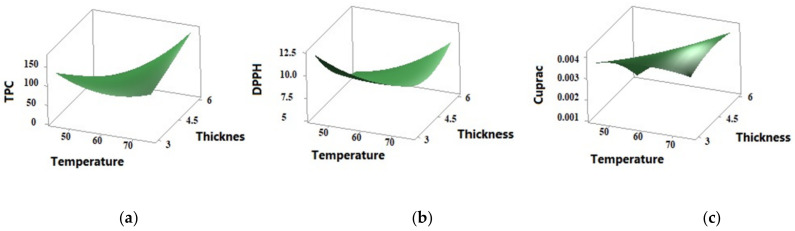
Surface plots of the antioxidant properties of the convectively dried sweet potatoes as affected by the drying conditions in terms of temperature and thickness: (**a**) TPC (mgGAE/g), (**b**) CUPRAC (mmolTR/g), (**c**) DPPH (% inhibition).

Table 2 shows the effect of temperature, time, thickness on the TPC, TFC, DPPH, and CUPRAC of the hot-air dried SP. The TPC value was positively affected by the linear and quadratic effect of temperature, whereas it was significantly negatively affected by time and thickness (*p* < 0.05). However, an increased TPC value was observed in terms of the quadratic effect of increasing time (Table 1 and Table 3). TPC was obtained as 193.5 mgGAE/g at a temperature of 75 °C after 4 h with a 6 mm-thick sample. Although potato is a food rich in phenolic components, the effect of temperature on the emergence of potato phenolic compounds was clearly observed. The effect of slice thickness as well as temperature on TPC values is clearly seen (Figure 4). The highest TPC value was detected in the freeze-dried PS (Table 5).

Flavonoids are important functional parts of phenolic compounds. Similarly to the change in TPC, a clear increase was observed in the TFC of sweet potatoes after convective drying (Table 4). The % inhibition of DPPH varied from 3.71 to 79.20% in the convectively dried SP. A higher DPPH scavenging activity of the freeze-dried samples was recorded. The drying factors did not affect the DPPH scavenging activity significantly (*p* < 0.05). The convectively dried PS have quite low CUPRAC values, varying from 1.0 to 4.7 µmol TR/g. However, the temperature of CD has a significant linear and quadratic effect on CUPRAC (*p* < 0.05). Although the CUPRAC value increased as the temperature increased, the value was found to be quite low compared to the freeze-dried SP. While there was no significant change in the anthocyanin content of the sweet potatoes dried by convective drying method at 45 and 60 °C, a significant decrease was observed in the dried samples at 75 °C (*p* < 0.05). No significant effect of time or thickness was identified. The optimum convective drying conditions for maximization of the anthocyanin content were determined as 63.78 °C, 4.78 h and 3 mm thickness (Table 5). Furthermore, the highest anthocyanin content was determined in freeze-dried samples. The change in anthocyanin content showed a positive correlation with TPC and TFC (*p* < 0.05).

### 3.5. Microstructure

**Figure 5:**
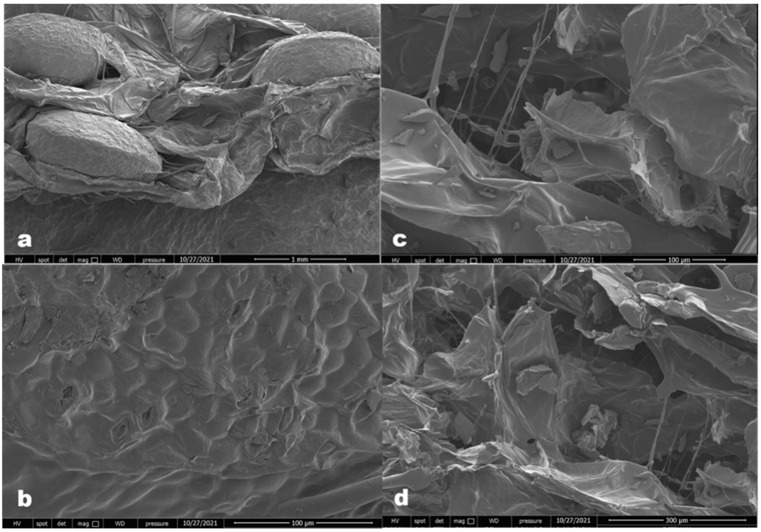
Scanning electron microscopy (SEM) images of: (**a**) hot air-dried SP (1 mm), (**b**) hot air-dried SP (100 µm), (**c**) freeze-dried SP (100 µm) and (**d**) freeze-dried SP (300 µm).

SEM images of hot air-dried and freeze-dried sweet potatoes are shown in Figure 5. A change in the polygonal cell structure of the sweet potato samples was observed as an effect of the applied drying processes. The cell wall disruptions and collapses are remarkable in the hot air-dried samples (Figure 5a,b). The evaporation caused by thermal flow increases intracellular stress and causes porosity in the CD method. Cell wall destruction and cellular spaces formed a spongy structure in the freeze-dried samples (Figure 5c,d). More cracked and more visual starch granules were determined in the freeze-dried samples compared to the SP dried using CD.

### 3.6. The Texture Characteristics

The textural properties of the dried sweet potatoes are summarized in Table 6. Higher values were observed for the mentioned properties in the convectively dried potatoes under optimum conditions, except for springiness. In terms of the general texture characteristics, CD gave higher values.

### 3.7. The Thermal Attributes

The thermal features are determined using differential calorimeters, which are the phenomena used to determine the interactive trends of food ingredients during food processing. The heating process affects the gelatinization, crystallization and amylation properties of foods with a high starch content. DSC is widely used to study the gelatinization and dissolution properties of crystalline starch species as well as starch retrogradation. The gelatinization endotherm achieved by DSC provides an overall measure of the gradual progressive loss of long, medium and short grades in crystallite starch granules. No endothermic peaks were determined for the hot air-dried samples under optimum conditions nor for the freeze-dried samples.

## 4. Discussion

### 4.1. Fitting of Model and Physicochemical Properties

The variances were constant for all the drying factors from the residual distribution around the line in the versus fit plots. The findings confirm that both the normal probability plot and the appropriate probability plot from experimental models for all responses are sufficient to describe the RSM used in this study.

Temperature has been reported as the most important factor that directly affects drying kinetics in conventional drying, while other factors such as air velocity, shape, size, and moisture content are also effective [21]. A decrease in the moisture content due to high temperature can lead to an undesirable color and appearance, especially if the moisture content is below 10% [6]. In our study, the decrease in water activity and moisture content depends on increasing temperature and decreasing slice thickness (Figure 2a,b). Slice thickness was reported to be an effective drying parameter regarding moisture content [12]. It is considered an important parameter in the drying process, as it directly affects the distance required for moisture diffusion, and the excessive decrease in thickness causes surface hardening, which prevents moisture diffusion [22].

Although the heating time did not show a significant linear and quadratic effect on the moisture content and water activity, the interactive effect of the heating time and the thickness was determined to be significant (*p* > 0.05) on the decrease in water activity (Table 3). The optimum temperature, time and slice thickness values used to minimize the moisture content and water activity were predicted as 63.79 °C, 4.79 h and 3.00 mm, respectively, from the model. The a_w_ and MC values were 0.059 and 0.5 g/100 g in the freeze-dried samples, respectively.

The shrinkage value is expressed as a quantity indicating moisture diffusivity [23]. In porous foods, the reduction in volume is higher as moisture diffusion through the pores occurs easily [24]. Some researchers have reported that temperature and air velocity did not have a significant effect on shrinkage, whereas greater shrinkage values of hot air-dried fruits and vegetables have been reported at low drying temperatures [12]. The moisture diffusion towards the sample surface increases as a result of increasing vapor pressure with the effect of increasing temperature. The most influential factor on the S_b_ value among all three factors was thickness. The shrinkage of the 3 mm-thick samples was significantly greater compared to the other samples. The thickness affects the distance for moisture diffusion, and, therefore, the fast-drying effect increases the shrinkage value of the surface. The shrinkage caused by surface drying and moisture degradation has been reported in potatoes [25]. The moisture diffusivity increases depending on the pre-treatment applied and interactive factors, such as the thickness and temperature [21]. Drying with dipso is used in many fruit chips to keep this value low. It is thought that the homogeneous distribution of color characteristics cannot be determined due to shrinkage in the samples, where the incompatibility of L*, a* and b* values with the hue angle and chroma value was determined. It has been reported that, as the slice thickness decreases, larger pores and a more porous structure are formed due to a decrease in the exit distance of the water, which undergoes phase change with the effect of heat [26]. While no volume change was experienced, the high rehydration capacity indicated the presence of passages formed by the sublimation of ice in the tissue during freeze drying. This phenomenon leads to preserving the matrix. The low ρ_a_ and ρ_b_ values found also support this.

### 4.2. Color Attributes

Color is a sensorial characteristic that significantly affects food preferability. Color attributes vary depending on pH, acidity, sugar and functional component ratios. The stability of color depends on the change in functional groups in the component composition. Thermal processes and process factors are also among the reasons for the change in functional components. Many drying processes activate applications that primarily stimulate moisture diffusion with thermal or non-thermal systems. Depending on the plant origin, pretreatment and combined drying methods affect the color properties. It is stated that the samples dried by vacuum drying gave lower lightness value than freeze-dried samples [27], ultrasound-pretreated sliced apples [28] and hot air-dried jujube [29]. Heating with temperature control before freeze-drying caused a decrease in the L* value in sliced avocados [10]. Karaman et al. [8] reported that freeze-dried persimmons were lighter in color than osmotically dehydrated samples. Regarding the color characteristics, this situation was associated with the inhibition of polyphenol oxidase depending on high temperature [30,31,32] and deterioration of pigments [28]. Prolonged drying times lead to a decrease in L*, a* and b* values by some chemical reactions such as enzymatic browning, the Maillard reaction and caramelization [17]. Hue angle and chroma value were also determined to observe the effect of the applications on color intensity. Temperature and time were the main factors affecting the hue angle and chroma values of the hot air-dried SP (Table 2 and Table 3). CD and FD increased the hue angle and decreased chroma values of fresh sweet potatoes. Similar results were reported for purple sweet potato powders dried by the drum drying method [17] and for infrared-dried sweet potatoes [21].

The color change of fresh to dried sweet potatoes was monitored according to the change in the ΔE values for the freeze and convective drying processes. The highest ΔE value was determined in freeze-dried samples at 26.9. The freeze drying process has been reported to provide a better appearance, with color properties compared to convective drying [33], and desired high L* values [10].

### 4.3. Antioxidant Activity

The phenolic compounds known as antioxidant sources in fruits and vegetables were reported to decrease with thermal processing [34]. However, it has also been reported that optimal processing conditions should be selected to ensure the antioxidant quality of fresh fruits and vegetables and their products, in order to preserve the activity and quality of natural health-promoting bioactive compounds, as well as to achieve the desired goals of a food processing method. Decreased TPC content was reported after the convective drying process in citrus fruit peels [35], grape seeds [36] and quinoa [6]. Alternatively, a significant positive effect of increasing the drying temperature on TPC values has been reported previously in tomato [3] and 5 mm-thick sliced hot air-dried sweet potato at 65 °C for 9 h [37]. As a result of the cellular destruction during the dehydration process that leads to an accelerated release of phenolic compounds, a greater amount of TPC in dried foods could be recorded [38]. The temperature-sensitive phenolic compounds will be degraded by the convective drying method, resulting in a decrease in total phenol content [39]. Conversely, it has been reported that this sensitivity does not occur with the freeze-drying process, so this situation is reflected in the amount of phenolic component [40]. Phenolic compounds and flavonoids with high thermal sensitivity are lost during convective drying [41]. The freeze-drying process allows these phenolic compounds to be dried by ice sublimation without being damaged. The flavonoids were more damaged from the convective drying method than from the freeze-, vacuum and infrared drying methods [8,35]. The flavonoid content could be negatively affected by the processing method and pretreatments in foods depending on the chemical matrix [14,42].

The results for DPPH scavenging and TAC were also similar to the results for TPC and TFC. Similar to our results, in many studies it has been reported that the freeze-drying method has provided higher DPPH removal activity [37] and TAC [43] than other methods, such as convective drying and microwave drying.

### 4.4. Microstructure and Textural Properties

Evaporation caused by thermal flow increases intracellular stress and causes porosity in the CD method. Previous data on the microstructure in dried sweet potato samples reported that convective drying creates more intracellular voids and a more porous structure when used in combination with other drying methods, such as infrared drying [26]. Cell wall destruction and cellular spaces formed a spongy structure in the freeze-dried samples (Figure 5c,d). More cracked and more visual starch granules were determined in the freeze-dried samples compared with those in the SP dried using CD. Larger pore sizes caused by the water separating from the intercellular space by sublimation positively affect the rehydration capacity of the dried samples. Similar to our results, a large and irregular porous structure formed by sublimation during freeze drying has been reported in potatoes [25]. The excess porosity that occurs in the freeze-drying process, besides increasing the rehydration speed due to the easier settlement of water in the pores caused by sublimation, unlike other drying methods, gave a structure without deformations [26].

The high porosity of freeze-dried samples affects their general properties, such as the rehydration capacity and shrinkage value, as well as the texture properties [43]. The formation of a softer and more brittle structure is associated with the presence of many pores. In many other drying methods, the structure deformation that occurs during the evaporation of water does not occur due to ice sublimation during freeze drying. Therefore, the structure properties closest to the fresh state are preserved.

### 4.5. The Thermal Attributes

The absence of an endothermic peak of phase changes, dehydration, reduction and degradation indicates that there is no change in the starch granulation and gelatinization in the potato structure. Observations confirm that the starch of dried sweet potato preserves its amorphous structure depending on the applied temperature. Similar results were reported for drum-dried purple sweet potato by Senevirathna et al. [17].

## 5. Conclusions

The drying conditions (temperature time and slice thickness) of sweet potato slices were optimized for the convective drying method using RSM. The model was fitted in terms of color properties, physicochemical properties and antioxidant capacity. Temperature showed negative linear and quadratic effects on general properties, such as water activity, moisture content and shrinkage value, as well as positive effects on apparent and bulk density. Higher L* and b* values were observed, whereas lower a* values were recorded as a linear and quadratic effect of temperature. The TAC values of convectively dried samples were higher than those of the undried and freeze-dried samples. The highest lightness value was recorded in freeze-dried samples. Freeze-dried sweet potatoes, which have the best appearance and structure, demonstrate high TFC and TPC values in terms of functionality. Furthermore, under the best conditions of the convective drying, an increase in functional aspects, such as TFC, TAC, TPC, DPPH and CUPRAC, can be observed.

## Figures and Tables

**Table 1:** Box–Behnken design of RSM and the experimental data obtained using dependent variables.

	Independent Variables			Response Variables																
Run No.	X_1_	X_2_	X_3_	a_w_	MC (%)	S_b_ (%)	R_c_ (g/g)	ρ_a_	ρ_b_	B_v_ (A_420_/ g)	L*	a*	b*	Ch	H	TAC (mg/100 g)	TFC (mg CE/g)	TPC (mg GAE/g)	DPPH (%inhi Bition)	CUPRAC (µmol TR/g)
1	45	2	4.5	0.934	46.90	75.00	2.60	1.65	0.87	0.020	54.3	27.7	37.3	46.40	53.30	0.85	0.09	78.30	12.13	3.8
2	75	2	4.5	0.479	02.00	60.00	3.12	0.95	0.93	0.029	71.6	22.9	32.9	40.10	55.20	5.00	0.36	86.31	7.41	2.2
3	45	6	4.5	0.770	16.10	60.00	2.62	0.675	0.56	0.025	70.8	21.5	27.5	34.90	52.00	1.30	0.25	3.60	4.38	1.0
4	75	6	4.5	0.304	00.60	75.00	3.13	0.905	0.89	0.042	62.3	27.2	37.7	41.60	54.10	1.05	0.09	114.3	11.12	4.7
5	45	4	3	0.800	16.00	78.57	3.12	1.26	1.06	0.022	64.7	21.0	25.1	32.70	50.10	3.30	0.48	108.9	12.13	3.6
6	75	4	3	0.437	00.60	53.86	3.21	0.53	0.52	0.038	67.9	24.2	38.2	45.20	57.70	10.31	0.03	101.7	10.78	4.0
7	45	4	6	0.802	19.00	75.00	2.43	1.59	1.28	0.015	63.6	21.6	26.1	33.90	50.40	1.70	0.23	27.72	5.39	0.9
8	75	4	6	0.431	00.50	60.00	3.25	0.97	0.96	0.037	66.2	26.6	36.1	44.80	53.70	2.5	0.14	193.5	10.78	4.0
9	60	2	3	0.806	09.10	83.33	2.84	2.64	2.39	0.027	72.3	23.2	31.6	37.20	52.30	11.37	0.072	68.58	8.76	2.9
10	60	6	3	0.564	01.00	85.21	3.10	6.80	0.98	0.033	71.4	22.1	33.1	39.80	56.30	3.70	0.13	85.50	11.79	3.8
11	60	2	6	0.967	18.80	64.24	2.81	2.20	0.18	0.030	70.6	21.5	28.3	35.50	52.70	4.70	0.25	20.61	3.71	0.9
12	60	6	6	0.494	00.30	89.47	2.73	2.70	2.69	0.036	73.3	20.7	31.0	37.30	56.20	10.01	0.05	7.20	11.79	3.9
13	60	4	4.5	0.479	11.20	66.66	2.96	1.25	1.11	0.034	65.1	22.3	26.7	34.80	50.00	2.25	0.09	79.20	7.75	3.6
14	60	4	4.5	0.479	11.20	66.66	2.96	1.25	1.11	0.034	65.1	22.3	26.7	34.80	50.00	2.25	0.09	79.20	7.75	3.6
15	60	4	4.5	0.479	11.20	66.66	2.96	1.25	1.11	0.034	65.1	22.3	26.7	34.80	50.00	2.25	0.09	79.20	7.75	3.6

X_1_: temperature (°C). X_2_: time. X_3_: thickness (mm). Water activity (a_w_), moisture content (MC), shrinkage (S_b_), rehydration capacity (R_c_), apparent density (ρ_a_), bulk density (ρ_b_), browning value (B_v_). L*, a* and b* values, Hue (H) and chroma (Ch). Total anthocyanin content (TAC), total flavonoid content (TFC), total phenol content (TPC), radical scavenging activity (DPPH) and cupric ion antioxidant capacty (CUPRAC).

**Table 2:** Adequacy of the models fitted for sweet potato.

Parameters	Fitted Models	R^2^	*p*-Value (Regression)	*p*-Value (Lack of Fit)
a_w_	a_w_ = 3.041 − 0.0270 X_1_ − 0.207 X_2_ − 0.359 X_3_ + 0.000117 X_1_*X_1_ + 0.02913 X_2_*X_2_ + 0.0499 X_3_*X_3_ − 0.00009 X_1_*X_2_ − 0.00009 X_1_*X_3_ − 0.0192 X_2_*X_3_	96.19	0.005	0.518
MC	MC = 114.1 − 3.46 X_1_ − 17.95 X_2_ + 29.1 X_3_ + 0.0154 X_1_*X_1_ + 0.434 X_2_*X_2_ − 2.51 X_3_*X_3_ + 0.2450 X_1_*X_2_ − 0.0344 X_1_*X_3_ − 0.867 X_2_*X_3_	95.39	0.008	0.644
S_b_	S_b_ = 194 + 1.61 X_1_ − 36.6 X_2_ − 41.8 X_3_ − 0.0286 X_1_*X_1_ + 1.82 X_2_*X_2_ + 2.95 X_3_*X_3_ + 0.250 X_1_*X_2_ + 0.108 X_1_*X_3_ + 1.95 X_2_*X_3_	79.02	0.215	0.300
L*	L = 1.7 + 2.785 X_1_ + 6.16 X_2_ − 16.32 X_3_ − 0.01478 X_1_*X_1_ + 0.744 X_2_*X_2_ + 1.700 X_3_*X_3_ − 0.2150 X_1_*X_2_ − 0.0067 X_1_*X_3_ + 0.300 X_2_*X_3_	98.31	0.001	0.000
a*	a = 71.1 − 1.431 X_1_ − 6.65 X_2_ + 2.49 X_3_ + 0.00889 X_1_*X_1_ + 0.131 X_2_*X_2_ − 0.422 X_3_*X_3_ + 0.0875 X_1_*X_2_ + 0.0200 X_1_*X_3_ + 0.025 X_2_*X_3_	84.27	0.121	0.000
b*	b = 118.5 − 2.097 X_1_ − 14.57 X_2_ − 2.53 X_3_ + 0.01672 X_1_*X_1_ + 0.847 X_2_*X_2_ + 0.406 X_3_*X_3_ + 0.1217 X_1_*X_2_ − 0.0344 X_1_*X_3_ + 0.100 X_2_*X_3_	83.49	0.134	0.000
TPC	TPC = 979 − 24.14 X_1_ + 28.4 X_2_ − 122.0 X_3_ + 0.1198 X_1_*X_1_ − 8.88 X_2_*X_2_ + 0.80 X_3_*X_3_ + 0.856 X_1_*X_2_ + 1.922 X_1_*X_3_ − 2.53 X_2_ + X_3_	91.26	0.034	0.337
DPPH	DPPH = 83.0 − 1.140 X_1_ − 7.43 X_2_ − 11.70 X_3_ + 0.00393 X_1_*X_1_ + 0.032 X_2_*X_2_ + 0.505 X_3_*X_3_ + 0.0955 X_1_*X_2_ + 0.0749 X_1_*X_3_ + 0.421 X_2_*X_3_	74.40	0.311	0.003
CUPRAC	CUPRAC = 0.01586 − 0.000152 X_1_ − 0.00229 X_2_ − 0.00183 X_3_ − 0.000001 X_1_*X_1_ − 0.000116 X_2_*X_2_ − 0.000117 X_3_*X_3_ + 0.000044 X_1_*X_2_ + 0.000030 X_1_*X_3_ + 0.000175 X_2_*X_3_	88.36	0.064	0.001
TAC	TAC = 22.6 − 0.05 X_1_ − 7.73 X_2_ − 1.0 X_3_ − 0.00256 X_1_*X_1_ + 0.942 X_2_*X_2_ + 0.000 X_3_*X_3_ + 0.0281 X_1_*X_2_ + 0.0378 X_1_*X_3_ − 0.246 X_2_*X_3_	46.85	0.834	0.518

X_1_: temperature (°C). X_2_: time. X_3_: thickness (mm). a_w_: water activity. MC: Moisture content. S_b_: shrinkage. Total phenol content (TPC), radical scavenging activity (DPPH), cupric ion antioxidant capacity (CUPRAC), total anthocyanin content (TAC).

**Table 3:** The *p* value and regression coefficient of the main, quadratic and interaction effects of different variables in the final reduced models fitted for sweet potato.

Response		Main Effects			Quadratic Effects			Interaction Effect		
		X_1_	X_2_	X_3_	X_1_^2^	X_2_^2^	X_3_^3^	X_1_X_2_	X_1_X_3_	X_2_X_3_
MC	*p*-value	0.001	0.005	0.382	0.190	0.481	0.057	0.020	0.738	0.289
	Coef	−11.8	−7.35	1.49	3.46	1.74	−5.64	7.35	−0.77	−2.60
a_w_	*p*-value	0.000	0.003	0.670	0.492	0.022	0.025	0.939	0.955	0.151
	Coef	−0.20	−0.13	0.01	0.026	0.116	0.112	−0.002	−0.002	−0.057
S_b_	*p*-value	0.143	0.289	0.615	0.187	0.144	0.176	0.123	0.574	0.208
	Coef	−4.96	3.39	−1.53	−6.43	7.27	6.63	7.50	2.43	5.84
R_c_	*p*-value	0.000	0.197	0.001	0.476	0.007	0.425	0.924	0.001	0.019
	Coef	0.242	0.026	−0.131	0.020	−0.112	0.022	−0.002	0.182	−0.085
B_v_	*p*-value	0.010	0.594	0.301	0.746	0.291	0.826	0.286	0.965	0.312
	Coef	0.0031	0.00043	−0.0008	0.0003	−0.0013	0.00026	0.0013	0.00005	0.0012
L*	*p*-value	0.005	0.033	0.436	0.003	0.003	0.001	0.000	0.793	0.158
	Coef	1.825	1.125	−0.325	−3.325	2.975	3.825	−6.450	−0.150	0.900
a*	*p*-value	0.085	0.413	0.982	0.051	0.532	0.279	0.017	0.576	0.924
	Coef	1.138	−0.475	−0.012	2.000	0.525	−0.950	2.625	0.450	0.075
b*	*p*-value	0.023	0.932	0.501	0.072	0.095	0.604	0.070	0.646	0.857
	Coef	3.61	−0.10	−0.81	3.76	3.39	0.91	3.65	−0.78	0.30
H	*p*-value	0.047	0.411	0.576	0.331	0.061	0.137	0.962	0.334	0.906
	Coef	1.863	0.638	−0.425	1.13	2.52	1.85	0.05	−1.07	−0.12
Ch	*p*-value	0.112	0.669	0.794	0.153	0.392	0.826	0.196	0.862	0.931
	Coef	2.98	−0.70	−0.43	3.83	2.13	0.53	3.25	−0.40	−0.20
ρ_a_	*p*-value	0.625	0.346	0.331	0.089	0.134	0.123	0.722	0.966	0.198
	Coef	−0.22	0.454	−0.470	−1.352	1.147	1.190	0.233	0.028	−0.917
ρ_b_	*p*-value	0.658	0.487	0.879	0.058	0.442	0.169	0.719	0.768	0.003
	Coef	−0.06	0.094	0.020	−0.451	0.154	0.296	0.067	0.055	0.980
TPC	*p*-value	0.009	0.551	0.148	0.082	0.036	0.891	0.084	0.015	0.554
	Coef	34.66	−5.40	−14.46	27	−35.5	1.8	25.7	43.2	−7.6
DPPH	*p*-value	0.419	0.351	0.147	0.516	0.924	0.410	0.065	0.224	0.347
	Coef	0.757	0.884	−1.474	0.88	0.13	1.14	2.86	1.69	1.26
CUPRAC	*p*-value	0.039	0.134	0.071	0.591	0.267	0.510	0.014	0.116	0.200
	Coef	0.0007	0.0004	−0.0005	−0.0002	−0.0004	−0.0002	0.0013	0.0006	0.0005
Coef	*p*-value	0.474	0.631	0.764	0.807	0.152	1.00	0.710	0.708	0.745
	Coef	−1.17	0.78	0.48	−0.57	3.77	0	0.84	0.85	−0.74

Moisture content (MC), water activity (a_w_), shrinkage (S_b_), rehydration capacity (R_c_). browning value (B_v_). Lightness (L*), redness (a*) and yellowness (b*) values. Hue (H) and chroma (Ch), apparent density (ρ_a_). bulk density (ρ_b_), total phenol content (TPC), radical scavenging activity (DPPH) and cupric ion reducing activity (CUPRAC).

**Table 4:** Optimum convective drying conditions and responses of sliced sweet potato.

Process Parameters	Target	Experimental Range		Optimum Value	Desirability
		Min	Max		
Temperature (°C)	Range	45	75	75	
Time (hour)	Range	2	6	4.42	
Thickness (mm)	Range	3	6	6	
	Responses			Predicted Values	0.71
a_w_	Minimum	0.304	0.967	0.36	
MC (%)	Minimum	0.3	46.9	(−)2.75	
B_v_ (A_420_/g)	Maximum	0.015	0.042	0.039	
TAC (mg/100 g)	Maximum	0.85	11.37	11.30	
TFC (mgCE/g)	Maximum	0.03	0.48	0.179	
TPC (mgGAE/g)	Maximum	3.6	193.5	150.0	
DPPH (%inhibition)	Maximum	3.71	12.13	12.52	
CUPRAC (µmolTR/g)	Maximum	0.090	0.470	0.469	

Water activity (a_w_), moisture content (MC), Browning value (B_v_), total anthocyanin content (TAC), total flavonoid content (TFC), total phenol content (TPC), Radical scavenging activity (DPPH) and cupric ion antioxidant capacty (CUPRAC).

**Table 5:** Effects of convective drying (CD) and freeze drying (FD) methods on physico-chemical, color and functional properties of sliced sweet potato.

Variables		Sliced Sweet Potato	
	Undried	FD	CD_opt_
Moisture (g/100 g)	74.2	0.5	16.23
a_w_	1	0.059	0.83
X_s_ (g/g)	0.258	0.995	0.833
X_w_ (g/g)	0.742	0.005	0.165
S_b_ (%)	-	0	88.35
ρ_a_ (g/g)	0.86	0.295	2.71
ρ_b_ (g/g)	0.22	0.294	2.44
R_c_ (g/g)	1.22	4.10	2.87
B_v_ (A_420_/g)	0.016	0.060	0.0263
TPC (mgGAE/g)	112.86	190.94	157.75
DPPH (%inhibition)	5.05	12.45	12.05
CUPRAC (µmol TR/g)	1.1	5.15	3.9
TAC (mg/100 g)	1.60	0.12	11.37
TFC (mgCE/g)	0.26	0.30	0.117
L*	53.8	77	69.24
a*	24.7	18.6	23.17
b*	35.8	23.8	32.31
Ch	43.5	30.2	33.66
H	55.5	52.1	53.28

ND: not detected, a_w_: water activity, X_S_: mass fraction of the solid, X_w_: mass fraction of water S_b_: shrinkage, R_c_: rehydration capacity, B_v_: browning value, ρ_a_: apparent density, ρ_b_: bulk density. Total phenol content (TPC), radical scavenging activity (DPPH) and cupric ion antioxidant capacity (CUPRAC). Lightness (L*), redness (a*) and yellowness (b*) values, Hue (H) and chroma (Ch).

**Table 6:** The texture parameters of dried sweet potato samples effected by rehydration processes.

Samples	Hardness	Springiness Resilience	Chewiness	Cohesiveness	Adhesiveness
FD	11.45	0.89 0.18	2.56	0.54	−7.58
CD_opt_	12.76	0.89 0.31	6.87	0.72	−3.67
*p*-value	0.0001	NS	0.004	0.0001	0.0001

FD: freeze-dried, CD_opt_: optimum conditions modelled by RSM for hot air drying, NS: non-significant.
